# Identification of a conserved JEV serocomplex B-cell epitope by screening a phage-display peptide library with a mAb generated against West Nile virus capsid protein

**DOI:** 10.1186/1743-422X-8-100

**Published:** 2011-03-06

**Authors:** En-Cheng Sun, Jing Zhao, Tao Yang, Ni-Hong Liu, Hong-Wei Geng, Yong-Li Qin, Ling-Feng Wang, Zhi-Gao Bu, Yin-Hui Yang, Ross A Lunt, Lin-Fa Wang, Dong-Lai Wu

**Affiliations:** 1 The Key Laboratory of Veterinary Public Health, Ministry of Agriculture, State Key Laboratory of Veterinary Biotechnology, Harbin Veterinary Research Institute, Chinese Academy of Agricultural Sciences, Harbin 150001, PR China; 2 Graduate School of Chinese Academy of Agricultural Sciences, Beijing 100081, PR China; 3 Beijing Institute of Microbiology and Epidemiology, Beijing 100071, PR China; 4 CSIRO Livestock Industries, Australian Animal Health Laboratory, Geelong, Victoria 3220, Australia

## Abstract

**Background:**

The West Nile virus (WNV) capsid (C) protein is one of the three viral structural proteins, encapsidates the viral RNA to form the nucleocapsid, and is necessary for nuclear and nucleolar localization. The antigenic sites on C protein that are targeted by humoral immune responses have not been studied thoroughly, and well-defined B-cell epitopes on the WNV C protein have not been reported.

**Results:**

In this study, we generated a WNV C protein-specific monoclonal antibody (mAb) and defined the linear epitope recognized by the mAb by screening a 12-mer peptide library using phage-display technology. The mAb, designated as 6D3, recognized the phages displaying a consensus motif consisting of the amino acid sequence KKPGGPG, which is identical to an amino acid sequence present in WNV C protein. Further fine mapping was conducted using truncated peptides expressed as MBP-fusion proteins. We found that the KKPGGPG motif is the minimal determinant of the linear epitope recognized by the mAb 6D3. Western blot (WB) analysis demonstrated that the KKPGGPG epitope could be recognized by antibodies contained in WNV- and Japanese encephalitis virus (JEV)-positive equine serum, but was not recognized by Dengue virus 1-4 (DENV1-4)-positive mice serum. Furthermore, we found that the epitope recognized by 6D3 is highly conserved among the JEV serocomplex of the Family *Flaviviridae*.

**Conclusion:**

The KKPGGPG epitope is a JEV serocomplex-specific linear B-cell epitope recognized by the 6D3 mAb generated in this study. The 6D3 mAb may serve as a novel reagent in development of diagnostic tests for JEV serocomplex infection. Further, the identification of the B-cell epitope that is highly conserved among the JEV serocomplex may support the rationale design of vaccines against viruses of the JEV serocomplex.

## Background

West Nile virus (WNV) is a positive-sense, single-stranded RNA virus of the family *Flaviviridae*, genus *Flavivirus*. It is a member of the Japanese encephalitis virus (JEV) serocomplex, which is comprised of several medically important viruses including WNV, JEV, Saint-Louis encephalitis virus (SLEV) and Murray Valley fever virus (MVEV) [1,2]. The close antigenic relationship of viruses belonging to the JEV serocomplex accounts for the serologic cross-reactivity seen in diagnostic laboratories.

The 10.7-kilobase WNV genome is translated into a single polyprotein, which is subsequently processed by viral- and host-encoded proteases into structural and nonstructural proteins. Three structural proteins (C, prM/M and E) make up the viral particle and seven nonstructural proteins (NS1, NS2A, NS2B, NS3, NS4A, NS4B and NS5) are required for genome replication and polyprotein processing [3].

The capsid (C) protein is the building block of the nucleocapsid. The C protein is a small 12 kD protein composed of 105 amino acids, and is highly positively charged due to a large number of lysine and arginine residues. The charged residues are clustered at the N- and C-terminal ends, and are separated by an extremely conserved internal hydrophobic region which mediates membrane association [4]. The nascent capsid protein also contains a C-terminal hydrophobic anchor that serves as a signal peptide for the endoplasmic reticulum translocation of the membrane precursor [5]. The secondary structure of recombinant C protein from Dengue virus (DENV) 2 and Yellow Fever virus(YFV), as determined by NMR techniques, shows that *flavivirus *C proteins are predominately dimeric in solution and are composed of four alpha helices (a1-a4), in which the N terminus (residues 1-20) is conformationally labile or unstructured [6]. The first elucidated 3 D structure of DENV C protein dimer (residues 21-100) suggested possible mechanisms for its interactions with RNA and the viral membrane [7].

*Flavivirus *C proteins are targeted by host immune responses. The specificities of a serotype-specific human CD4+ cytotoxic T-lymphocyte clone (CTL) and a panel of serotype cross-reactive human CD4+ CTL have been mapped to epitopes contained within the DENV4 C protein, indicating that anti-viral T cell responses are directed against C protein-derived peptides [8]. Further, the production and characterization of anti-DENV C antibodies suggests that the N terminus region covering the first 20 amino acids of DENV C protein is the predominant target of humoral immune responses in mice [9].

The aim of our study was to identify WNV-specific and/or JEV serocomplex-specific B-cell epitopes on C protein using phage display technology. Phage display has proven to be a powerful and economic technique for epitope identification and has been used widely in epitope mapping in flaviviruses [10-13]. The results described in this report will facilitate the development of diagnostic tests for the specific serological evaluation of WNV/JEV serocomplex infection and further understanding of the antigenic structure of C protein which will benefit the rationale design of JEV serocomplex vaccines.

## Results

### Production of recombinant C protein

**Figure 1:**
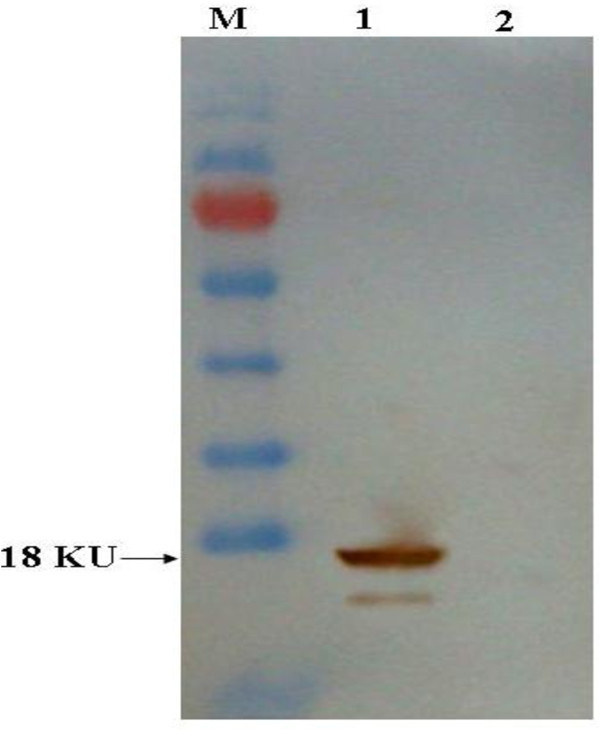
**Recombinant C protein is recognized by antibodies in WNV-positive equine serum**. Purificated recombinant C protein (lane 1) and cell lysates from insect cells infected with wild-type baculovirus (lane 2) were evaluated for reactivity with serum from WNV-positive horses. M: PageRuler™ Prestained Protein Ladder (Fermentas, Canada).

The recombinant WNV C protein used as antigen for monoclonal antibody generation was considered firstly. A baculovirus expression system was used to produce recombinant WNV C protein in Sf9 insect cells. The recombinant C protein generated in insect cells was recognized by antibodies contained in WNV-positive equine serum by Western blot (WB) (Figure 1).

### Production and characterization of C protein-specific mAb

**Figure 2:**
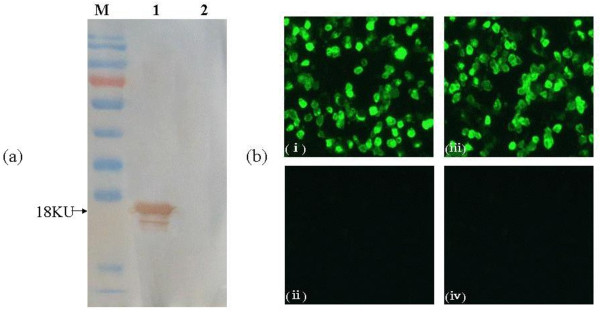
**The mAb 6D3 recognizes recombinant C protein and WNV-infected C6/36 cells**. (a) Recombinant C protein (lane 1) and cell lysates from insect cells infected with wild-type baculovirus (lane 2) were evaluated for reactivity with mAb 6D3 by WB. M: PageRuler™ Prestained Protein Ladder (Fermentas, Canada). (b) Analysis of immunofluourescence produced by (i) mAb 6D3, (ii) a negative control mAb (anti-porcine IFN-γ mAb), (iii) a positive control serum (WNV-positive mouse serum), and (iv) a negative control serum (WNV-negative mouse serum) on acetone-fixed, WNV infected C6/36 cells (WNV antigen slides).

Purified C protein was used to immunize BALB/c mice. After cell fusion and screening, several hybridoma cell lines were generated which produced C-reactive mAbs. Among them, the antibody produced by the line designated as 6D3 was selected for strong reactivity against recombinant C protein in WB (Figure 2, panel a) and in an indirect ELISA (data not shown). The 6D3 mAb also showed strong reactivity against WNV antigen slides by an indirect immunofluorescence assay (IFA; Figure 2, panel b). The 6D3 mAb recognized the JEV serocomplex viruses WNV and JEV by IFA, while no reactivity against the non-JEV serocomplex flaviviruses DENV1-4, YFV and Tick-borne encephalitis virus (TBEV) was seen (data not shown).

The 6D3 mAb was composed of an IgG1 heavy chain paired with a λ-type light chain, as determined using the Mouse MonoAb-ID Kit (HRP). The titer of antibody in hybridoma cell culture supernatants and in ascities was measured by indirect ELISA and was determined to be 1:512 and 1:1,024,000, respectively.

### Phage enrichment by biopanning

The purified 6D3 mAb was used to pan a phage displayed peptide library to determine the fine specificity of the C protein-specific mAb. After three rounds of biopanning, a marked enrichment of phages was achieved from the phage displayed 12-mer library. The output to input ratio following each of the three rounds of biopanning was 0.00018%, 0.024% and 0.89%.

### Epitope prediction

**Figure 3:**
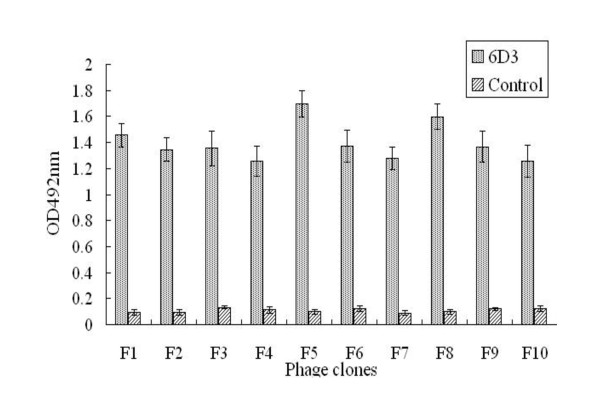
**The mAb 6D3 specifically recognizes clones selected from the phage display peptide library**. Ten clones selected after three rounds of biopanning of a phage display peptide library used the 6D3 mAb by phage-ELISA. The anti-porcine IFN-γ mAb served as a negative control.

**Figure 4:**
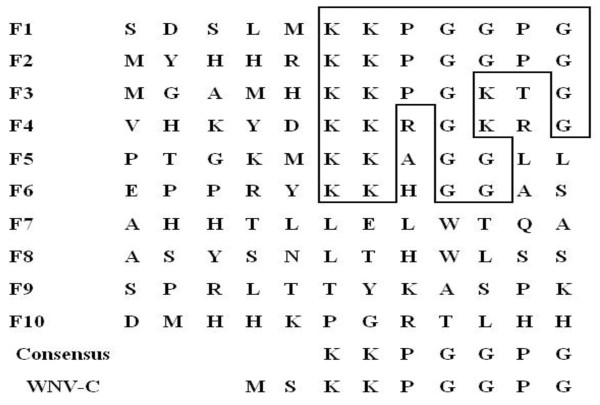
**Alignment of 12-mer peptide sequences from ELISA-positive clones defines a linear epitope for the mAb 6D3**. The peptide insert from ten phage clones that reacted with the mAb 6D3 were aligned. Conserved amino acid residues are boxed and the consensus sequence motif is provided below the alignment. The matching sequence _3_KKPGGPG_9 _of the WNV C protein is provided at the bottom of alignment for comparison.

Ten phage clones were selected for reactivity with 6D3 following enrichment of the phage display peptide library. These selected clones were further evaluated by ELISA for reactivity with the 6D3 mAb and a negative control mAb (anti-porcine IFN-γ). As shown in Figure 3, the 6D3 mAb reacted with each clone (F1-F10), giving optical density readings at 492 nm (OD492 nm) greater than 1.0. In contrast, the negative control antibody gave low OD492 nm readings (OD492 nm < 0.2). These data indicate that the 6D3 mAb specifically reacts with the ten phage clones that were selected following three rounds of enrichment of the peptide library with 6D3. We next sequenced the peptide insert of the ten selected phage clones that reacted with the 6D3 mAb. An alignment of the peptide insert sequences indicated that six 6D3-reactive clones (F1-F6) displayed a consensus peptide sequence of KKPGGPG. The consensus sequence motif defined by the peptide library screen are identical to the sequence _3_KKPGGPG_9 _found in WNV C protein (Figure 4), indicating that peptide library screen successfully identified the C protein epitope recognized by 6D3.

### Fine mapping of epitope

**Figure 5:**
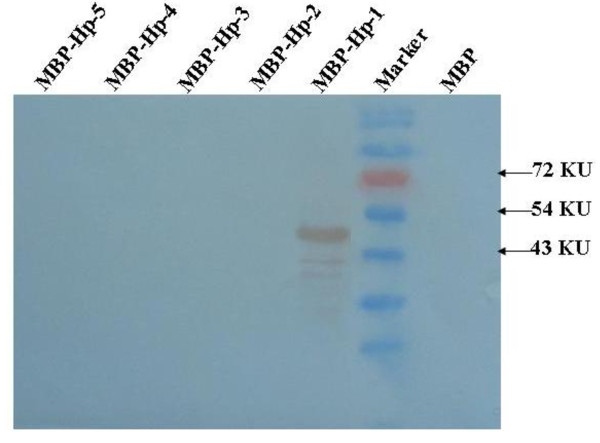
**KKPGGPG is the minimal epitope recognized by the mAb 6D3**. Full length and truncated versions of the KKPGGPG epitope were expressed as MBP fusion proteins and assessed for reactivity with the mAb 6D3 by WB. MBP-Hp-1, KKPGGPG; MBP-Hp-2, KPGGPG; MBP-Hp-3, KKPGGP; MBP-Hp-4, PGGPG; MBP-Hp-5, KKPGG; M: PageRuler™ Prestained Protein Ladder (Fermentas, Canada).

For further epitope determination, we generated a series of truncated peptides derived from the KKPGGPG peptide that was identified by screening the peptide library with the 6D3 mAb. The full-length and truncated peptides (Table 1) were generated as MPB fusion proteins and were used in WB analysis with the 6D3 mAb. We found that only the full-length KKPGGPG polypeptide (MBP-Hp-1) was recognized by mAb 6D3 (Figure 5). Removal of one or more amino acids at either the amino or carboxy terminus of the polypeptide abolished antibody binding, indicating that the polypeptide KKPGGPG is the minimal linear epitope recognized by 6D3 (Figure 5).

**Table 1:** The oligonucleotides encoding full-length and truncated versions of the KKPGGPG motif

Designations of oligonucleotides	The sequences of oligonucleotides	Coding motifs (designations)
Hp-1-F	5'-AATTCaagaaaccaggagggcccggcTAAG-3'	KKPGGPG (Hp-1)
Hp-1-R	5'-TCGACTTAgccgggccctcctggtttcttG-3'	
Hp-2-F	5'-AATTCaaaccaggagggcccggcTAAG-3'	KPGGPG (Hp-2)
Hp-2-R	5'-TCGACTTAgccgggccctcctggtttG-3'	
Hp-3-F	5'-AATTCaagaaaccaggagggcccTAAG-3'	KKPGGP (Hp-3)
Hp-3-R	5'-TCGACTTAgggccctcctggtttcttG-3'	
Hp-4-F	5'-AATTCccaggagggcccggcTAAG-3'	PGGPG (Hp-4)
Hp-4-R	5'-TCGACTTAgccgggccctcctggG-3'	
Hp-5-F	5'-AATTCaagaaaccaggagggTAAG-3'	KKPGG (Hp-5)
Hp-5-R	5'-TCGACTTAccctcctggtttcttG-3'	

***Notes*: **Bases introduced to form the overhanging ends of *EcoR *I and *Sal *I after annealing the two complementary oligonucleotides are shown in capital letters; stop codons TAA are added.

### WNV- and JEV-positive serum reactivity with the identified epitope

**Figure 6:**
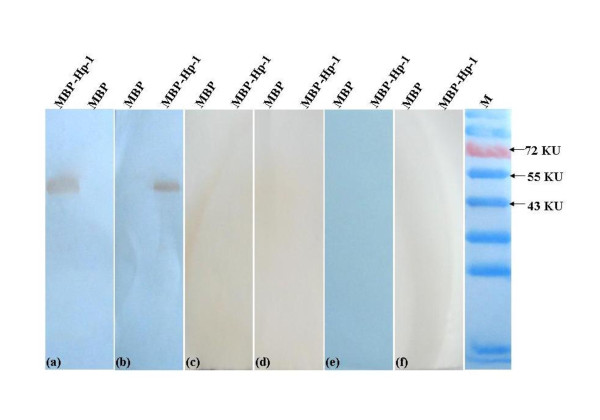
**Antibodies in WNV/JEV-positive equine sera recognize the MBP fusion protein containing the KKPGGPG epitope**. MBP alone or MBP fused with the KKPGGPG peptide epitope (MBP-Hp-1) was evaluated by WB analysis for reactivity with antibodies in (a) WNV-positive equine serum, (b) JEV-positive equine serum, (c) DENV1-positive mouse serum, (d) DENV2-positive mouse serum, (e) DENV3-positive mouse serum, and (f) DENV4-positive mouse serum. M, PageRuler™ Prestained Protein Ladder (Fermentas, Canada).

To assess whether the minimal linear epitope was immunogenic in the context of JEV serocomplex infection, we tested WNV- and JEV-positive equine serum for antibodies specific for the KKPGGPG polypeptide expressed as an MBP fusion protein (MBP-Hp-1). Serum from WNV-positive horses (Figure 6, panel a) and JEV-positive horses (Figure 6, panel b) reacted with the MBP-Hp-1 fusion protein containing the KKPGGPG epitope, but not with MBP protein alone. Serum from DENV1-4 positive mice did not react with the MPB-Hp-1 fusion protein (Figure 6, panels c-f). These data were further confirmed by ELISA (data not shown). These results demonstrate that the minimal linear B-cell epitope is targeted by humoral immune responses in the context of bona fide JEV serocomplex virus infection.

### Sequence similarity and prediction of cross-reactivity

**Figure 7:**
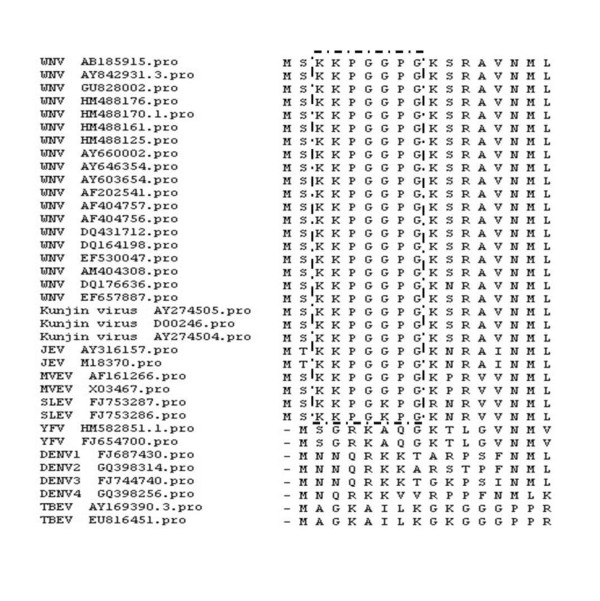
**Alignment of WNV and other flavivirus C protein amino acid sequences surrounding the 6D3 epitope region**. Amino acids from the region encompassing the mAb 6D3 epitope of 22 WNV strains (including 3 Kunjin virus strains) and 14 other flavivirus strains were aligned for analysis using Lasergene analysis software. The sequence motif recognized by the mAb 6D3 is boxed.

We then evaluated the conservation of the KKPGGPG epitope among viruses of the JEV serocomplex. Analysis of C protein sequences from 28 different JEV serocomplex isolates demonstrates that the epitope recognized by 6D3 is conserved among the JEV serocomplex, with the exception of SLEV C protein, in which a G-to-K mutation is found (Figure 7). The motif is absent in non-JEV serocomplex members of *Flaviviridae *family (Figure 7).

## Discussion

Monoclonal antibodies with well defined epitopes have become a powerful tool to study protein structure and have been widely used to diagnose and treat a variety of infectious agents [14-18]. The binding properties of antibodies can be used experimentally to define antigenic structures of pathogen-associated proteins and to understand virus-antibody interactions at a molecular level. In this study, we described the generation and epitope mapping of a WNV C-protein-specific mAb, and demonstrate that the epitope is conserved among many JEV serocomplex members. Precise analysis of WNV C protein epitopes will lead to a better understanding of host immune responses, the development of epitope-based marker vaccines, and diagnostic tools for WNV and/or JEV serocomplex infection.

Phage display is an *in vitro *selection technique in which a peptide or protein is genetically fused to a coat protein of bacteriophage, resulting in display of the fused protein on the exterior of the phage virion. A phage display library can consist of either random peptide libraries or gene-targeted libraries, thus providing a powerful and economic technique for epitope identification. This technology can identify amino acids on protein antigens that are critical for antibody binding and, further, can define peptide motifs that are both structural and functional mimotopes of both protein and non-protein antigens [19,20].

In our current study, we generated a C protein specific mAb, named 6D3, using recombinant C protein expressed in insect cells by a recombinant baculovirus system. We found that the 6D3 mAb reacted with WNV and JEV by IFA, but not with other non-JEV serocomplex flaviviruses, such as DENV1-4, YFV and TBEV. The linear epitope recognized by the 6D3 mAb was defined as KKPGGPG using phage display technology to perform a screen of a peptide library. This peptide sequence directly corresponded to a region of WNV C protein with the sequence _3_KKPGGPG_9_. Further fine mapping using truncation mutants revealed the core determinant of the mAb binding site was KKPGGPG. The peptide was also recognized by WNV/JEV-positive equine serum, indicating that the epitope is immunogenic in horses in the context of viral infection. Consistent with analysis of cross reactivity using IFA and WB with WNV/JEV-positive equine and DENV1-4-positive mouse serum, sequence alignments of JEV serocomplex sequences demonstrated that the motif is highly conserved among JEV serocomplex members, but is absent in other viruses of the *Flavivirus *genus.

## Conclusions

We have generated the C protein-specific 6D3 mAb and shown that it recognizes a linear epitope that is highly conserved among the JEV serocomplex. The 6D3 mAb has great potential to improve JEV-serocomplex diagnostic tests and aid the design of robust epitope-based vaccines.

## Methods

### Cell lines, plasmid and serum specimens

The myeloma cell line SP2/0 was cultured in Dulbecco's modified Eagle's medium (DMEM, Invitrogen) in a humidified 5% CO_2 _atmosphere at 37°C. All culture media were supplemented with 10% heat-inactivated fetal bovine serum (GIBCO, Invitrogen), 0.1 mg/ml of streptomycin and 100 IU/ml of penicillin. The WNV NY99 genome (GenBank accession number AY842931.3) was cloned into plasmid pMAL™ -C2x (New England Biolabs, Inc., USA), and JEV-positive/negative equine serum and DENV1-4-positive/negative mouse serum were maintained in our laboratory. WNV-positive/negative mouse serum was obtained from the Beijing Institute of Microbiology and Epidemiology, and the WNV-positive equine serum was from the CSIRO Australian Animal Health Laboratory (AAHL).

### Expression of recombinant C protein

Recombinant WNV C protein was prepared according to the product instructions of the Bac-to-Bac^® ^Baculovirus Expression System (Invitrogen). In brief, the C gene from WNV NY99 strain was cloned into the pFastBac™ vector. The recombinant pFastBac™ vector was then transformed into competent DH10Bac™ *E. coli *cells, which were subsequently plated on triple antibiotic LB plates (Kanamycin, Gentamicin and tetracycline) with BluoGal. The site-specific transposition reaction takes place between the mini-Tn7 elements (pFastBac™ vector) and the mini-attTn7 attachment sites on the bacmid DNA in DH10Bac™. This reaction is mediated by a transposase, an enzyme encoded by the helper plasmid that is also in DH10Bac™ *E. coli*. This transposition step disrupts the lacZ reading frame and allows blue/white screening. Colonies containing the recombinant bacmid DNA appear white, while colonies containing the non-recombinant bacmid DNA appear blue. Bacmid DNA was recovered from white colonies and was subsequently verified via PCR. Insect cells were transfected with recombinant Bacmid DNA by using Cellfectin^®^. Recombinant baculovirus supernatant was harvested 2-5 days after transfection, and was titered using the BaculoTiter™ Assay Kit according to manufacturer's instructions. Recombinant protein was analyzed by sodium dodecyl sulfate-polyacrylamide gel electrophoresis (SDS-PAGE) and purificated by Ni-nitrilotriacetic acid affinity chromatography (Qiagen) according to the manufacturer's instructions, then identified by WB. For WB, recombinant C protein and Sf9 cells infected with wild-type baculovirus were subjected to electrophoresis on 10% SDS-PAGE after reduction with dithiothreitol (DTT) at 100°C for 5 min. Samples were transferred to a nitrocellulose membrane and were blocked overnight with 5% skim milk powder in PBST at 4°C. The membrane was incubated with WNV-positive equine sera as the primary antibody, followed by an HRP-conjugated rabbit anti-equine secondary antibody (LICOR Biosciences). The color was developed using 3,3'-diaminobenzidine tetrahydrochloride (DAB) substrate and was stopped by rinsing in deionized water followed by drying the membrane.

### Preparation and characterization of mAbs against C protein

Hybridomas secreting C protein-specific antibodies were generated according to standard procedures with a few modifications [21]. Briefly, six-week-old female BALB/c mice were immunized subcutaneously with purified C protein emulsified with an equal volume of Freund's complete adjuvant (Sigma, St. Louis, MO, USA). Two booster injections containing purified C protein in Freund's incomplete adjuvant were given at 2-week intervals. A final immunization, consisted of purified C protein without adjuvant and was injected intraperitoneally. Three days after the final immunization, mice were euthanized, and spleen cells were harvested and fused with SP2/0 myeloma cells at 5-10:1 ratio using polyethylene glycol (PEG 4000, Sigma). The hybridoma cells were seeded into 96-well plates and selected in HAT medium (DMEM containing 20% fetal bovine serum, 100 ug/ml streptomycin, 100 IU/ml penicillin, 100 mM hypoxanthine, 16 mM thymidine and 400 mM aminopterin), and after 5 days, the medium was removed and replaced with fresh HT-DMEM medium. After HAT/HT selection, culture supernatants of surviving clones were screened for reactivity and specificity by indirect ELISA, WB and IFA.

The ELISA assay has been described previously [22]. Briefly, microplates were sensitized at 4°C overnight with the affinity-purified WNV-C protein at 50 ng/ml. The sensitized plates were incubated with test culture supernatants from hybridomas at 37°C for 1 h, with HRP-conjugated goat anti-mouse secondary antibodies (LICOR Biosciences) at a 1:4,000 dilution at 37°C for 1 h, followed by color development with substrate solution containing *o*-phenylenediamine (OPD).

WB was performed using mAbs as primary antibodies and a HRP-conjugated goat anti-mouse secondary antibody.

The IFA results were supplied by Beijing institute of Microbiology and Epidemiology. WNV, JEV, DENV1-4, YFV and TBEV antigen slides were prepared on porous slides using WNV, JEV, DENV1-4, YFV and TBEV infected and uninfected C6/36 cells. Cell suspensions were dripped onto slides, fixed using acetone, air-dried, and were stored at -20°C until use. Next, anti-C protein mAbs and WNV-, JEV-, DENV1-4-, YFV- and TBEV-positive/negative mouse serum (positive/negative control) were incubated on acetone-fixed antigen slides for 2 h. A FITC-conjugated goat anti-mouse IgG was used as a secondary antibody, and slides were viewed at a magnification of ×40 on a fluorescence microscope (Leica, Germany).

The positive clones were subcloned three times by limiting dilution. Selected clones were cultured in the peritoneal cavities of pristine-primed BALB/c mice to obtain ascites fluid.

The mAb titer was determined by indirect ELISA as described above and the antibody subtype was determined using the Mouse MonoAb-ID Kit (HRP) (Invitrogen, Carlsbad, CA, USA) according to the manufacturer's instructions. This test identifies the IgG1, IgG2a, IgG2b, IgG3, IgA and IgM subtype classes and the κ and λ light chains using monospecific rabbit polyclonal antibodies (Pabs).

### Determination of epitopes by phage-displayed random peptide library

The Ph.D.-12™ Phage Display Peptide Library Kit was purchased from New England BioLabs Inc. The dodecapeptide library consists of 2.7×10^9 ^electroporated sequences (1.5×10^13 ^pfu/ml). All of the mAbs were purified from the ascites fluid of mice inoculated with the hybridoma cells by affinity chromatography using rProtein G (Sigma, USA) according to the manufacturer's instructions. The concentration of purified protein was determined by the Bradford Protein Assay Kit (http://www.beyotime.com/ Compatibility Chart For Bradford Kit. Pdf).

Three successive rounds of biopanning were carried out according to the manufacturer's instructions (New England BioLabs Inc). Briefly, one well of a 96-well microtiter plate was coated with 15 μg of purified mAb in coating buffer (0.1 M NaHCO_3_, pH 8.6), followed by blocking with blocking buffer (0.1 M NaHCO3, pH8.6, and 5 mg/ml BSA) for 2 h at 4°C. About 1.5×10^11^pfu (4×10^10 ^phages, 10 μl of the original library) were added to the well and incubated for 1 h at room temperature by gentle shaking. The unbound phages were removed by successive washings with TBS buffer (50 mM Tris-HCl, pH7.5, 150 mM NaCl) containing gradually increasing concentrations of Tween-20 (0.1%, 0.3%, and 0.5%). The bound phages were eluted with elution buffer (0.2 M Glycine-HCl, 1 mg ml^-1 ^BSA, pH 2.2). The eluted phages were amplified in early-log *E. coli *ER2738 cells.

After three rounds of biopanning, ten individual phage clones were selected and assayed for target binding by sandwich ELISA as described by the manufacturer's instructions (New England BioLabs Inc). Briefly, 96-well microtiter plates were coated overnight with 2 μg of the 6D3 mAb or antiporcine IFN-γ mAb (Sigma, USA), which served as a negative control. After 2 h of blocking with blocking buffer at 4°C, phage clones were added to the wells (2×10^11^pfu in 100 μl per well) and incubated with agitation for 2 h at room temperature. Bound phages were subjected to reaction with HRP-conjugated anti-M13 antibody (Pharmacia, USA) for 2 h at room temperature, followed by color development with substrate solution containing *o*-phenylenediamine (OPD).

The DNA inserts displayed by ELISA-positive phage clones were sequenced with the 96 gIII sequencing primer: 5'-TGAGCGGATAACAATTTCAC-3' as described by the manufacturer's instructions (New England BioLabs Inc).

### Fine mapping of the epitope by WB

A series of complementary oligonucleotides (Table 1) encoding for the full-length and truncated versions of the peptide motif KKPGGPG were synthesized, annealed, and cloned into *EcoR *I/*Sal *I sites of prokaryotic expression vector pMAL™-C2x (New England Biolabs, Inc., USA), resulting in five recombinant plasmids. The *E. coli *TB1 cells transformed with the recombinant plasmids were induced with 0.5 mM IPTG to produce recombinant MBP-fused polypeptides. The series of MBP-fused polypeptides was screened by WB using the C protein-specific mAb as described above.

### Reactivity of the epitope with WNV/JEV-positive equine serum and DENV1-4-positive mouse serum

To test whether the KKPGGPG epitope could be detected by antibodies generated by a mammalian host in the context of WNV/JEV infection, we evaluated the reactivity of WNV/JEV-positive equine serum against MBP-Hp-1 (MBP fusion containing the peptide of KKPGGPG) by WB. WB was performed as described above, using a HRP-conjugated rabbit anti-equine secondary antibody (LICOR Biosciences). To test whether non-JEV serocomplex virus infection can induce antibodies specific for the KKPGGPG epitope, we evaluated the reactivity of DENV1-4-positive mouse sera against MBP-Hp-1 by WB, using an HRP-conjugated goat anti-mouse secondary antibody.

### Homology analysis

To investigate the conservation of the epitope among flaviviruses, sequence alignment of the epitope and amino acid sequences from the corresponding region on C protein of 22 WNV strains (including 3 Kunjin virus strains) was performed using the DNASTAR Lasergene program (Windows version; DNASTAR Inc., Madison, WI). Alignment analysis was also performed between the identified epitope and other associated flavivirus strains, including the members of JEV serocomplex, and another three antigenically related flavivirus, DENV1-4, YFV and TBEV. Factors related to the time of virus isolation and geographic region of origin of all strains were considered.

## Competing interests

The authors declare that they have no competing interests.

## Authors' contributions

DLW designed the experiment. ECS and JZ carried out most of the experiments and ECS wrote the manuscript. RAL supplied the equine sera against WNV. YHY supplied the mouse sera against WNV, JEV, DENV, YFV and TBEV. ZGB supplied the WNV C gene. TY, HWG, YLQ, LFW and NHL participated part of experiments. DLW and LFW revised the manuscript. All authors read and approved the final manuscript.

## References

[B1] Weissenbock H Kolodziejek J Url A Lussy H Rebel-Bauder B Nowotny N Emergence of Usutu virus, an African mosquito-borne flavivirus of the Japanese encephalitis virus group, central Europe Emerg Infect Dis 2002 8 652 656 12095429 10.3201/eid0807.020094 PMC2730324

[B2] Heinz FXCM Purcell RH Gould EA Howard CR Houghton HM Moormann RJM Rice CM Thiel HJ Family Flaviviridae San Diego, CA: Virus taxonomy 7th report of the international committee for the taxonomy of viruses 2000

[B3] Castillo-Olivares J Wood J West Nile virus infection of horses Vet Res 2004 35 467 483 10.1051/vetres:2004022 15236677

[B4] Mandl CV Heinz FX Kunz C Sequence of structural proteins of tickborne encephalitis virus (western subtype) and comparative analysis with other flaviviruses Virology 1988 166 197 205 10.1016/0042-6822(88)90161-4 3413985

[B5] Dokland T Walsh M Mackenzie JM Khromykh AA Kim-Huey E S Wang West Nile virus core protein: tetramer structure and ribbon formation Structure 2004 12 1157 1163 10.1016/j.str.2004.04.024 15242592 PMC7173237

[B6] Jones CT Ma L Burgner JW Groesch TD Post CB Kuhn RJ Flavivirus capsid is a dimeric alpha-helical protein J Virol 2003 77 7143 7149 10.1128/JVI.77.12.7143-7149.2003 12768036 PMC156156

[B7] Ma L Jones CT Groesch TD Kuhn RJ Post CB Solution structure of dengue virus capsid protein reveals another fold Proc Natl Acad Sci 2004 101 3414 3419 10.1073/pnas.0305892101 14993605 PMC373476

[B8] Gagnon SJ Zeng W Kurane I Ennis FA Identification of two epitopes on the dengue 4 virus capsid protein recognized by a serotypespecific and a panel of serotype-cross-reactive human CD4+ cytotoxic T-lymphocyte clones J Virol 1996 70 141 147 8523518 10.1128/jvi.70.1.141-147.1996 PMC189798

[B9] Puttikhunt C Ong-ajchaowlerd P Prommool T Sangiambut S Netsawang J Limjindaporn T Malasit P Kasinrerk W Production and characterization of anti-dengue capsid antibodies suggesting the N terminus region covering the first 20 amino acids of dengue virus capsid protein is predominantly immunogenic in mice Arch Virol 2009 154 1211 1221 10.1007/s00705-009-0426-5 19565324

[B10] Bugli F Mancini N Kang CY Di Campli C Grieco A Manzin A Gabrielli A Gasbarrini A Fadda G Varaldo PE Clementi M Burioni R Mapping B-cell epitopes of hepatitis C virus E2 glycoprotein using human monoclonal antibodies from phage display libraries J Virol 2001 75 9986 9990 10.1128/JVI.75.20.9986-9990.2001 11559832 PMC114571

[B11] Zhang F Yu M Zhang N Wang LF Characterization of epitopes for neutralizing monoclonal antibodies to classical swine fever virus E2 and Erns using phage-displayed random peptide library Arch Virol 2006 151 37 54 10.1007/s00705-005-0623-9 16132176

[B12] Herrmann S Leshem B Lobel L Bin H Mendelson E Ben-Nathan D Dussart P Porgador A Rager-Zisman B Marks RS T7 phage display of Ep15 peptide for the detection of WNV IgG J Virol Methods 2007 141 133 140 10.1016/j.jviromet.2006.11.041 17215048

[B13] Peng Wu-Ping Hou Qiang Xia Zhao-He Chen Dan Li Na Sun Yuan Qiu Hua-Ji Identification of a conserved linear B-cell epitope at the N-terminus of the E2 glycoprotein of Classical swine fever virus by phage-displayed random peptide library Virus Res 2008 135 267 272 10.1016/j.virusres.2008.04.003 18485511

[B14] Sukupolvi-Petty Soila Austin S Kyle Engle Michael Brien James D Dowd Kimberly A Williams Katherine L Johnson Syd Rico-Hesse Rebeca Harris Eva Pierson Theodore C Fremont Daved H Diamond Michael S Structure and Function Analysis of Therapeutic Monoclonal Antibodies against Dengue Virus Type 2 J Virol 2010 84 9227 9239 10.1128/JVI.01087-10 20592088 PMC2937608

[B15] Shen Xiaoying Parks Robert J Montefiori David C Kirchherr Jennifer L Keele Brandon F Decker Julie M Blattner William A Gao Feng Weinhold Kent J Hicks Charles B Greenberg Michael L Hahn Beatrice H Shaw George M Haynes Barton F Tomaras Georgia D In Vivo gp41 Antibodies Targeting the 2F5 mAb Epitope Mediate HIV-1 Neutralization Breadth J Virol 2009 83 3617 3625 10.1128/JVI.02631-08 19193787 PMC2663270

[B16] Denisova Galina F Denisov Dimitri A Yeung Jeffrey Loeb Mark B Diamond Michael S Bramson Jonathan L A novel computer algorithm improves antibody epitope prediction using affinity-selected mimotopes: A case study using monoclonal antibodies against the West Nile virus E protein Mol Immunol 2008 46 125 134 10.1016/j.molimm.2008.07.020 18760481 PMC3856767

[B17] Levy R Forsyth CM LaPorte SL Geren IN Smith LA Marks JD Fine and Domain-level Epitope Mapping of Botulinum Neurotoxin Type A Neutralizing Antibodies by Yeast Surface Display J Mol Biol 2007 365 196 210 10.1016/j.jmb.2006.09.084 17059824 PMC1994578

[B18] May Chad Doody Jacqueline F Abdullah Rashed Balderes Paul Xu Xiaohong Chen Chien Peter Zhu Zhenping Shapiro Lawrence Kussie Paul Hicklin Daniel J Liao Fang Bohlen Peter Identification of a transiently exposed VE-cadherin epitope that allows for specific targeting of an antibody to the tumor neovasculature Blood 2005 105 4337 4344 10.1182/blood-2005-01-0010 15701713

[B19] Rowley MJ O'Connor K Wijeyewickrema L Phage display for epitope determination: a paradigm for identifying receptor-ligand interactions Biotechnol Annu Rev 2004 10 151 188 full_text 15504706 10.1016/S1387-2656(04)10006-9

[B20] Wang LF Yu M Epitope identification and discovery using phage display libraries: applications in vaccine development and diagnostics Curr Drug Targets 2004 5 1 15 10.2174/1389450043490668 14738215

[B21] Kohler G Milstein C Continuous cultures of fused cells secreting antibody of predefined specificity Nature 1975 256 495 497 10.1038/256495a0 1172191

[B22] Konishi E Fujii A Mason PW Generation and characterization of a mammalian cell line continuously expressing Japanese encephalitis virus subviral particles J Virol 2001 75 2204 2212 10.1128/JVI.75.5.2204-2212.2001 11160724 PMC114804

